# Supplementary material for: Societal cost of day-care approach (DCA): A low-cost approach in urban and rural settings for management of childhood severe pneumonia in Bangladesh
Source: PLoS One. 2025 May 8;20(5):e0323353. doi: 10.1371/journal.pone.0323353 (PMC12061116; doi:10.1371/journal.pone.0323353)
Supplement: Attachment — Submitted filename: ResponseTOReviewer_R1.docx [file pone.0323353.s002.docx]

**Response to Reviewer Comments**

**Manuscript ID - PONE-D-24-44140**

**Title:** Societal cost of day-care approach (DCA): a low-cost approach in urban and rural settings for management of childhood severe pneumonia in Bangladesh

| **Response to the Academic Editor's and Reviewer’s Comments** | | | |
| --- | --- | --- | --- |
| The authors thank the editor and reviewer for their time taken to provide valuable feedback, and for the opportunity to resubmit the paper addressing their comments. | | | |
| **Academic Editor’s comments and suggestions** | | **Author’s response** | **Changes to document (in the revised cleaned version)** |
| Thank you for submitting your manuscript to PLOS ONE. After careful consideration, we feel that it has merit but does not fully meet PLOS ONE’s publication criteria as it currently stands. Therefore, we invite you to submit a revised version of the manuscript that addresses the points raised during the review process. | | | |
| **1** | The topic says “societal costs of DCA”…  What does societal costs mean? | The authors thank the editor for reviewing the paper. We have added the definition of societal cost considered in this study. | ***Method section***  *Page 7, lines 137 to 146 that now read as:*  ***“Perspective***  *The perspective is crucial in conducting an economic evaluation of a health intervention, as it defines the scope of the study and determines which costs and outcomes are included in the analysis [26]. There are various types of perspectives including the patient, healthcare provider, health system, and societal [26]. The societal perspective includes all healthcare-related costs, regardless of the payer, such as patients' costs (e.g., travel, accommodation). It may also encompass non-health-related impacts in sectors such as productivity loss of the caregiver [27]. This study adopted a societal perspective to capture all costs borne by households (including caregiver productivity loss) and providers for managing one episode of childhood severe pneumonia in DCA.”* |
| **2** | Even though it says societal costs, what is presented in the findings is economic costs. What makes it different from economic costs, which have already been studied and confirmed feasible? | Thank you once again for your valuable comments. While the costs considered in our study may encompass economic costs, we have now included additional details to clarify the definition of societal costs used in this study.  Furthermore, we have added a reference that outlines the various perspectives commonly used in cost analysis and economic evaluations. Since our study accounts for all costs related to the treatment procedure as well as the productivity loss of caregivers, we have categorised these as societal costs. | Relevant text in the method section has been added in page 7 lines 141 to 146:  “*The societal perspective includes all healthcare-related costs, regardless of the payer, such as patients' costs (e.g., travel, accommodation). It may also encompass non-health-related impacts in sectors such as productivity loss of the caregiver [27]. This study adopted a societal perspective to capture all costs borne by households (including caregiver productivity loss) and providers for managing one episode of childhood severe pneumonia in DCA.”* |
| **3** | The feasibility for DCA is already confirmed to be feasible in Bangladesh. You mentioned this in previous RC studies. So, what was the importance of your study? What value will it add or which gap will it fill? | Thank you for your detailed review. The previous RCT focused on severe pneumonia and severe malnutrition and was conducted as small-scale pilot studies in urban-based tertiary settings (n=120), where the cost per patient was estimated. In contrast, this effectiveness trial was implemented across multiple primary public healthcare facilities in a broader context, involving 32 facilities and over 3,200 participants (DCA and UC). This day-care approach is particularly novel and significant for policymakers in Bangladesh, as it aims to reduce 24-hour hospitalisations, addressing the limited availability of beds to treat all children in public health facilities.  We have added additional details to the introduction (rationale) section in the revised manuscript. | Introduction section  Page 5 lines 94 to 101.  *“Two randomized trials compared severe pneumonia DCA management to hospital care [21,22], both concluding that when timely referral mechanisms are in place, severe pneumonia can be successfully managed in DCA (~95% cases) with lower costs compared to hospital care. However, these small-scale pilot studies were limited to the capital city of Bangladesh (an urban setting) with limited sample sizes, focusing primarily on the clinical effectiveness of the new DCA approach. As a result, gaps remain in understanding the relative costs and cost-effectiveness of this innovative intervention within existing public healthcare settings in Bangladesh covering both urban and rural contexts. To test the DCA model in a broader range, including urban and rural primary-level facilities, a cluster randomized effectiveness trial was conducted by the International Centre for Diarrhoeal Disease Research, Bangladesh (icddr,b) to assess the clinical effectiveness of DCA compared to usual care (UC) in the real-life setting.”* |
| **4** | One of the findings indicates Rural setting is more feasible for DCA than an urban setting, right? How could you justify this finding in scientific ways? Is that because of certain biases (e.g. urban people pay more than rural people do or ????), better if you can justify them scientifically? | We agree with the reviewers' concerns. One of the aims of the trial was to assess the clinical effectiveness and cost of the DCA model in rural area since inpatient care is not available in most rural primary level facilities. Many studies have highlighted forgone healthcare seeking in rural settings due to the unavailability of inpatient facilities and other barriers such as transportation and caregiver constraints.  In this study, although we showed that urban implementation is more costly (as expected), we demonstrated that the intervention is feasible in rural areas. Moreover, we provided a policy recommendation indicating that implementing this approach in rural settings would be less costly for the government, significantly improve healthcare access, and save children’s lives by preventing them from being left untreated at home.  We have added the relevant text to the discussion section of the revised manuscript. | **Discussion section**  Page 21 lines 381 to 382; lines 384 to 391.  *“In our study, the mean per patient cost of the DCA model shows that travel costs and caregiver time/productivity costs are lower using the model for childhood severe pneumonia management in both urban and rural located facilities, compared to the available published literature on inpatient care for severe pneumonia [21,22,24,25]. This highlights the strong argument that implementing the DCA model could significantly reduce both supply and demand-side barriers in both urban and rural areas. While the costs for medicine and supportive care to treat children with severe pneumonia may be similar across urban and rural settings, implementing this approach in rural areas would be less costly for the government, would substantially improve healthcare access, and could save children’s lives by preventing them from being left untreated at home due to demand and supply-side barriers. In addition, addressing this disparity by strengthening rural healthcare services could also promote more equitable access to care while potentially reducing the reliance on costly inpatient treatments if manageable by DCA settings.”* |
| **5** | The way the indirect costs and opportunistic costs were valued/given price is not clear. | Thank you for the comment. We have added detailed text explaining the meaning of indirect costs in our study. | **Method section**  Page 7 lines 151 to 158 now reads as:  *“In estimating household costs, both direct medical costs, direct non-medical costs, and indirect costs were considered. Direct medical costs included out-of-pocket (OOP) expenses for resources such as consultations and medicines, while direct non-medical costs covered non-healthcare items like transport used for treatment purposes. Indirect costs were estimated using the human capital approach, multiplying lost working hours by the self-reported hourly wage rate, as per previous studies in similar settings, including Bangladesh [4,24,33]. For caregivers not engaged in paid work, the minimum government-fixed hourly wage rate was applied [24]. Data to estimate caregivers’ indirect costs, including waiting time and lost productivity or opportunity costs, were collected through a structured questionnaire.”* |
| **6** | In the statistical analysis part, p values only indicate significance level. But, the direction of the association is not clear. E.g. what indicates if cost is higher among urban compared to rural? | Thank you for your comment. We have expanded the results section, particularly in the statistical analysis subsection (regression analysis), to provide greater detail. | **Result section**  **Page 17 lines 294 to 298.**  *“An increase of one additional treatment day in was associated with a 7% increase in log-transformed total cost (p < 0.001). Belonging to the ‘Richer’ wealth quintile was associated with a statistically significant 5% increase in costs compared to the ‘Poorest’. Considering facility location, residing in rural areas was associated with a statistically significant 12% decrease in costs compared to urban areas (p < 0.001). The presence of malnutrition in children with severe pneumonia was not significantly associated with increased costs.”* |
| **7** | You mentioned as the you collected both qualitative and quantitative data. So, what type of qualitative data, to answer what and where are the findings from qualitative data | Thank you for the comment. Authors apologise for the unclear sentence. What we meant to say is that previous literature identified barriers to seeking care using both qualitative and quantitative methods, and our study showed that the DCA model could help address those barriers.  However, this study did not use a qualitative approach. We have revised the text to clarify this. | **Discussion section**  **Page 21, lines 373, 374, 379-382 now read as:**  *“Previous published evidence highlighted the significance of financial and non-financial impediments in seeking care for under-five children in LMICs adopting both qualitative and quantitative research and suggests that accessibility and financial barriers, such as long waiting times, long distance to a healthcare facility, lack of transport, high OOP expenses, socio-economic and socio-cultural position are associated with delayed/abandoned care-seeking, specifically in rural areas [8,9,30,31,37,38].*  *In our study, the mean per patient cost of the DCA model shows that travel costs and caregiver time/productivity costs are lower using the model for childhood severe pneumonia management in both urban and rural located facilities, compared to the available published literature on inpatient care for severe pneumonia [21,22,24,25]***.** |
|  | **Reviewer’s comments and suggestions** |  |  |
|  | Dear authors, Thank you. This manuscript undertakes a straightforward analysis of ‘Societal cost of day-care approach (DCA): a low-cost approach in urban and rural settings for management of childhood severe pneumonia in Bangladesh’. There are a few little fixes that would, in my view, improve the manuscript for "consumption" by other researchers. This is the new, innovative and important research in developing country context. | | |
| **1** | In an economic sense: median cost was more effective than mean. If you include both the mean and median cost, I think you should be included in the median and mean cost. | We agree with the reviewer's concern. We have added the median value with IQR in the results section for the main cost components. However, we did not include this in the tables for all variables, as many of the median values were zero, which would have made the tables cluttered. | **Result section in page 13 has now been re-written as**  *‘The estimated mean societal cost per patient per episode of childhood severe pneumonia treated with DCA model was US$94.2 (95% CI: 92.2, 96.3) [median (Interquartile Range (IQR)): 84.5 (33.5)], with a mean length of stay of 4.1 days (Table 3). The largest contributor to cost was personnel cost (mean: US$ 32.6, median US$24.8), accounting for 35% of the total cost, followed by caregiver's time cost (27%) [mean: US$ 25.8; median: US$ 20.4) and cost for medicines (23%). The mean healthcare provider cost per patient was US$63.3 (95% CI: 62.4, 64.4), [median (IQR): 57.3 (22.8)] while the mean household cost per patient was US$30.9 (95% CI: 29.3, 32.5) [median (IQR): 24.2 (14.2)].”* |
| **2** | Why did you suggest DCA intervention is low cost? What do you mean by resource- poor setting in pneumonia management? Are urban and rural facilities truly comparable? Otherwise, urban and rural costs might be significantly different? | Thank you for the detailed review and suggestions. We have revised the discussion section to provide a clearer description of our findings. | **Discussion sections.**  Page 21, lines **379-382**  *In our study, the mean per patient cost of the DCA model shows that travel costs and caregiver time/productivity costs are lower using the model for childhood severe pneumonia management in both urban and rural located facilities, compared to the available published literature on inpatient care for severe pneumonia [21,22,24,25]***.**  Page 21, lines 384 to 391, now read as:  *This highlights the strong argument that implementing the DCA model could significantly reduce both supply and demand-side barriers in both urban and rural areas. While the costs for medicine and supportive care to treat children with severe pneumonia may be similar across urban and rural settings, implementing this approach in rural areas would be less costly for the government, would substantially improve healthcare access, and could save children’s lives by preventing them from being left untreated at home due to demand and supply-side barriers. In addition, addressing this disparity by strengthening rural healthcare services could also promote more equitable access to care while potentially reducing the reliance on costly inpatient treatments if manageable by DCA settings.* |
| **3** | Please see the line- 104 to 106. Your estimation might be focused on cost effectiveness analysis. But not focused on low-cost interventions. | Thank you for spotting this issue. We have rewritten the lines to make the study objective clearer. | **Introduction section**  Page 5, lines 107-108; Page 6, lines 110-113  *“To understand the detailed cost of the intervention arm of the trial (DCA approach), it is inevitable to comprehensively measure the per-patient cost of DCA arm, enabling a comparison of both treatment approaches to determine the low cost treatment option As such, in the present study, a societal perspective was adopted* *to estimate per patient cost of DCA, as well as to examine cost variation by facility locations (urban-rural), socio-demographic and clinical characteristics, to determine cost predictors. The findings of the study will inform clinical trialists and policymakers in determining whether the new approach is a low-cost solution that improves access to care, particularly for rural residents, and has a broader impact on improving care-seeking.”* |
| **4** | Please see the line- 111 to 114. Please describe the true comparable facilities of DCA and UC? | As our focus was solely on the DCA approach, we have included the intervention description and referred to the clinical trial paper for further details. In the trial, all DCA and UC participants were recruited from primary-level outpatient or inpatient facilities. However, they were referred for further care if oral antibiotic treatment failed within 48 hours. | No changes made within the paper. |
| **5** | Please see the line- 136 to 143. All direct costs should not be OOPE. When do you estimate the opportunity cost in provider perspectives? Please describe annuitization of capital items and how you should estimation using inflation adjustment. | Thank you for the suggestion. We have incorporated more details on the method of costing in the revised version of the manuscript. | **Method of costing section now reads as**  **“***In estimating household costs, both direct medical costs, direct non-medical costs, and indirect costs were considered. Direct medical costs included out-of-pocket (OOP) expenses for resources such as consultations and medicines, while direct non-medical costs covered non-healthcare items like transport used for treatment purposes. Indirect costs were estimated using the human capital approach, multiplying lost working hours by the self-reported hourly wage rate, as per previous studies in similar settings, including Bangladesh. For caregivers not engaged in paid work, the minimum government-fixed hourly wage rate was applied. Data to estimate caregivers’ indirect costs, including waiting time and lost productivity or opportunity costs, were collected through a structured questionnaire.*  *To estimate provider costs, we developed a comprehensive list of all resources used in one episode of care, including capital and recurrent/variable cost items such as personnel, healthcare consumables, and non-healthcare consumables. Capital item is defined as the items that is usually invested in bulk and used over time; that is, for more than one year, and recurrent item is defined as the items that are purchased regularly within a year. We then measured individual-use and shared-use items. To determine the unit cost for each resource, we used the price of the item or estimated opportunity cost. For instance, capital resource items are used over multiple years, and therefore, their costs were spread over time. The equivalent annual cost (EAC) for each capital item was calculated to determine the annual cost for each asset used by the provider. EAC was derived based on the quantity, unit price, and assumed useful lifetime of each asset (5–10 years for equipment and 30 years for buildings), as indicated in the literature and warranty periods. Data collection tools minimized double counting by collecting patient-specific data from household and provider perspectives, ensuring accurate expense allocation and cross-checking for potential duplicates. Further details of methods of measuring and valuing household and provider cost items can be found in our previous publications.^24,25”^* |
| **6** | In provider cost, you should be change in variable cost. I think it is the recurrent cost. In provider cost, I have not seen the training and meeting related cost? How do you consider opportunity cost in provider perspective? | We have incorporated the reviewer’s suggestion in the revised version. We did not include training and meeting costs, as they were solely considered research-related costs for the trial. We have added relevant text in the appropriate section for clarity. | **The section now reads as**  *‘To estimate the societal cost of DCA, household costs and healthcare provider costs were combined for each child. Staff training in this study was integrated into the research-related costs associated with the trial setting and, therefore, was not included in the patient-level cost analysis. The analysis considered resource use from the period of trial enrolment date to the three–month post-discharge follow-up period, therefore discounting was not applied. ‘* |
